# Critical Limb Ischaemia in a Patient With Subcutaneous Nodules: Clues to a Hidden Systemic Disease

**DOI:** 10.7759/cureus.99204

**Published:** 2025-12-14

**Authors:** Vasanth Kannan, David Lopez-Lazaro, Sampreety Majumder, Tharun Adithya Natesan, Cornelius Fernandez James

**Affiliations:** 1 Internal Medicine, United Lincolnshire Hospitals NHS Trust, Boston, GBR; 2 Physiology, Barind Medical College Hospital, Rajshahi, BGD; 3 Endocrinology and Metabolism, Pilgrim Hospital, Boston, GBR

**Keywords:** acute limb ischaemia, iatrogenic calcinosis cutis, nxp-2 antibody, s: calciphylaxis, s: dermatomyositis

## Abstract

Dermatomyositis most commonly presents with characteristic rash or proximal muscle weakness; however, specific autoantibody subsets can produce markedly atypical phenotypes. Anti-NXP2 dermatomyositis is clinically significant for its strong association with malignancy, extensive calcinosis, and presentations that may be amyopathic or minimally symptomatic. We report a rare case of anti-NXP2-positive dermatomyositis manifesting without classical cutaneous or muscular features, instead presenting with acute limb ischemia and long-standing subcutaneous nodules. Amyopathic anti-NXP2 disease is uncommon in adults, accounting for a small minority of dermatomyositis cases, making this presentation particularly unusual. Imaging demonstrated diffuse subcutaneous and intramuscular calcifications with large-vessel disease, while serologic testing confirmed anti-NXP2 antibodies. Following revascularization and multidisciplinary evaluation, limb perfusion and wound healing improved for this case. Amyopathic presentations of anti-NXP2 dermatomyositis are rare, reported in only a small subset of patients, highlighting the atypical nature of this case. This case underscores the need to consider autoimmune myositis in patients presenting with calcinosis cutis and ischemic changes, as recognition of anti-NXP2 dermatomyositis is essential for early diagnosis, multidisciplinary management, guidance of oncologic surveillance, and prevention of irreversible vascular outcomes.

## Introduction

Calcinosis cutis is a rare condition characterized by abnormal deposition of calcium salts in the skin and subcutaneous tissue. Although uncommon, it is a significant clinical finding frequently linked to autoimmune diseases such as systemic sclerosis, dermatomyositis, systemic lupus erythematosus, and primary Sjögren’s syndrome. It may also occur secondary to metabolic derangements or tissue injury. First described by Virchow in 1855, calcinosis cutis affects 18-49% of patients with systemic sclerosis and is seen in at least 30% of adults with dermatomyositis and 20-40% of juvenile cases [1]. Clinical features range from firm dermal papules and plaques to painful nodules causing functional limitation or ulceration. Diagnosis relies on clinical assessment supported by laboratory evaluation and imaging [2].

Calcinosis cutis is broadly classified into dystrophic, metastatic, idiopathic, iatrogenic, and calciphylaxis-related forms. Dystrophic calcinosis cutis is the most common type, caused by dermal damage or abnormalities, such as alterations in collagen, elastin, or subcutaneous fat. This condition is associated with normal calcium and phosphate levels [3]. Calciphylaxis represents a severe vasculopathy characterized by medial arterial calcification and thrombosis, most commonly in advanced renal disease, and carries a high mortality rate [3,4]. Differentiating between these entities is crucial, as their clinical implications and management differ substantially.

The anti-NXP2 autoantibody subset is strongly associated with extensive calcinosis and an increased risk of malignancy. Importantly, calcinosis cutis may serve as an early clue to underlying autoimmune myopathy [5]. This case highlights that anti-NXP2-positive dermatomyositis can present predominantly with calcinosis cutis and even limb-threatening ischemia, despite the absence of classical cutaneous or muscular features. This atypical presentation highlights the importance of early clinical suspicion and comprehensive evaluation, as it poses significant management challenges due to limited response to pharmacological therapies, including warfarin, probenecid, diltiazem, bisphosphonates, and colchicine, and may necessitate surgical intervention [6].

## Case presentation

**Figure 1:**
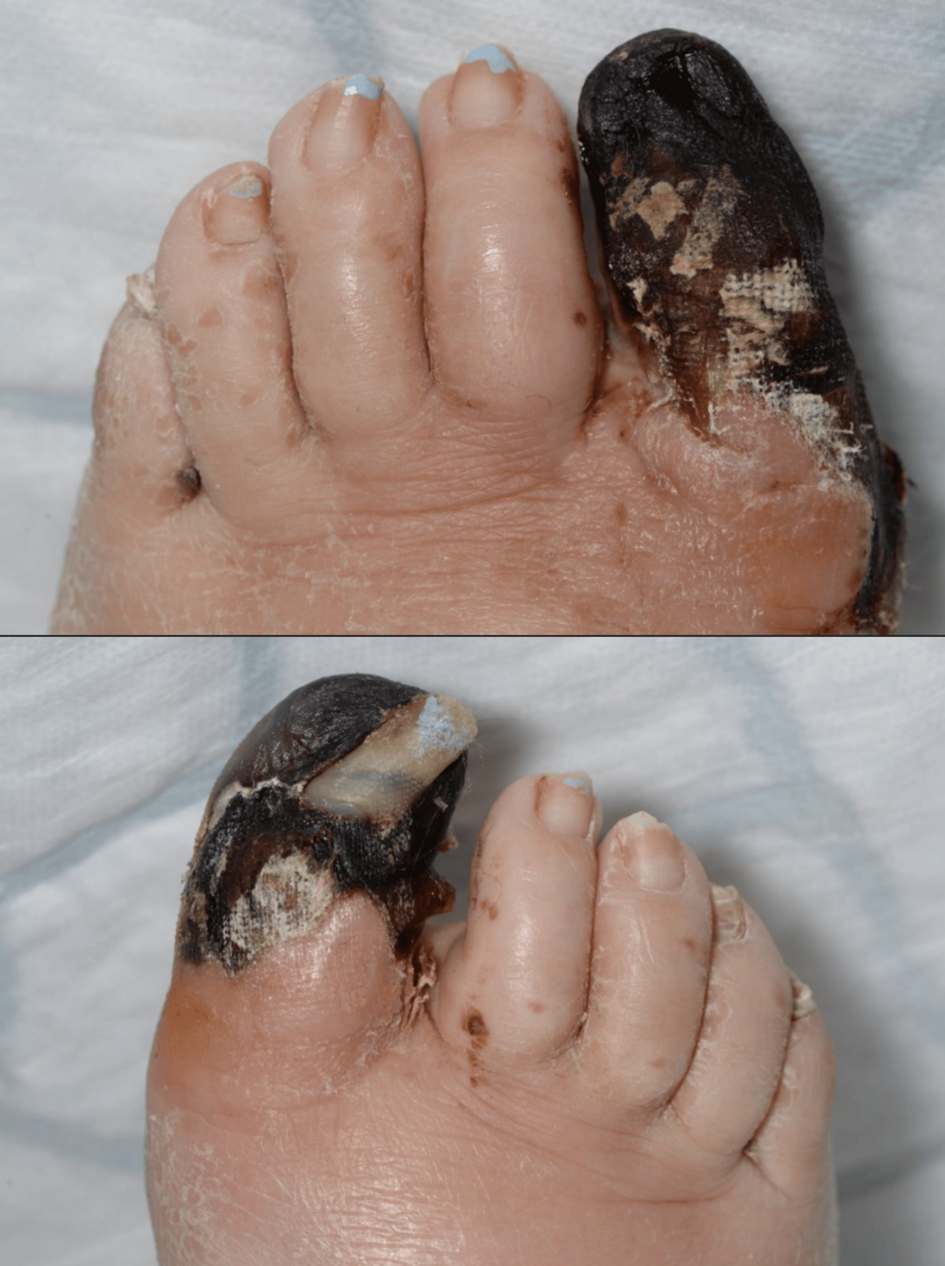
Clinical imaging showing bilateral first toe gangrene. Consent was taken from the patient for medical photography.

**Figure 2:**
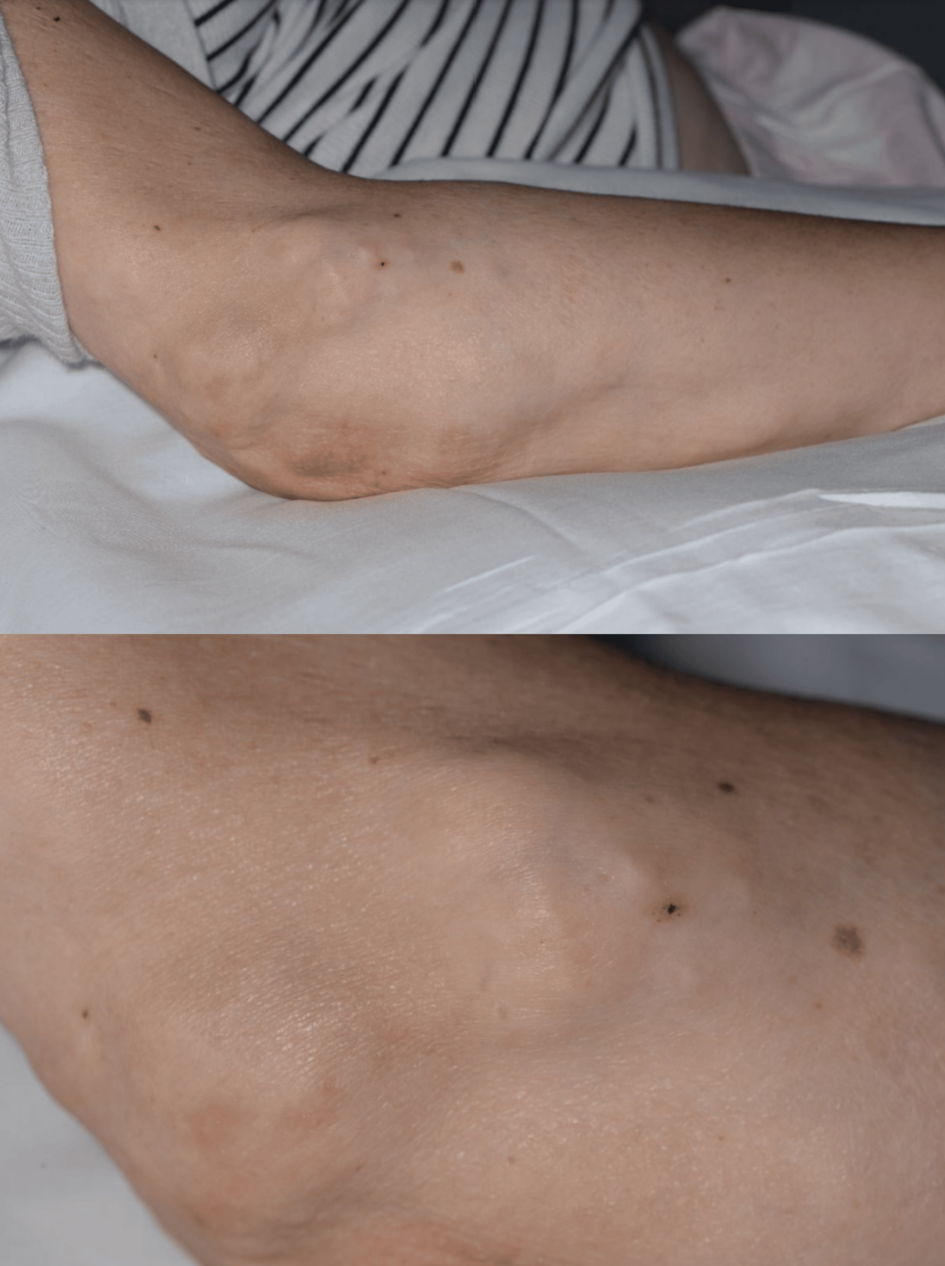
Clinical image showing multiple, firm to hard, non-tender and non-mobile subcutaneous nodules measuring approximately 0.5 to 1.5 cm in diameter over both elbows. Consent was taken from the patient for medical photography.

A 43-year-old woman presented with a two-week history of bilateral first toe discolouration, black in nature, associated with swelling and pain. Her past medical history included a single episode of pancreatitis in 2006. The symptoms began shortly after a pedicure, two to three weeks before admission, initially affecting the left hallux, which started to turn dark, gradually over four to five days. This was followed by involvement of the right hallux. This prompted her to attend the Accident and Emergency department. Examination revealed palpable dorsalis pedis pulsations. There was a presence of bilateral first toe infection with gangrene, as shown in Figure 1. Multiple hard nodules were noted over both elbows, abdomen, and thighs, resembling calcifications, as shown in Figure 2. Also, the arterial Doppler clinical imaging investigation of this patient was suggestive of bilateral monophasic waveforms and absent posterior tibial artery signals.

**Figure 3:**
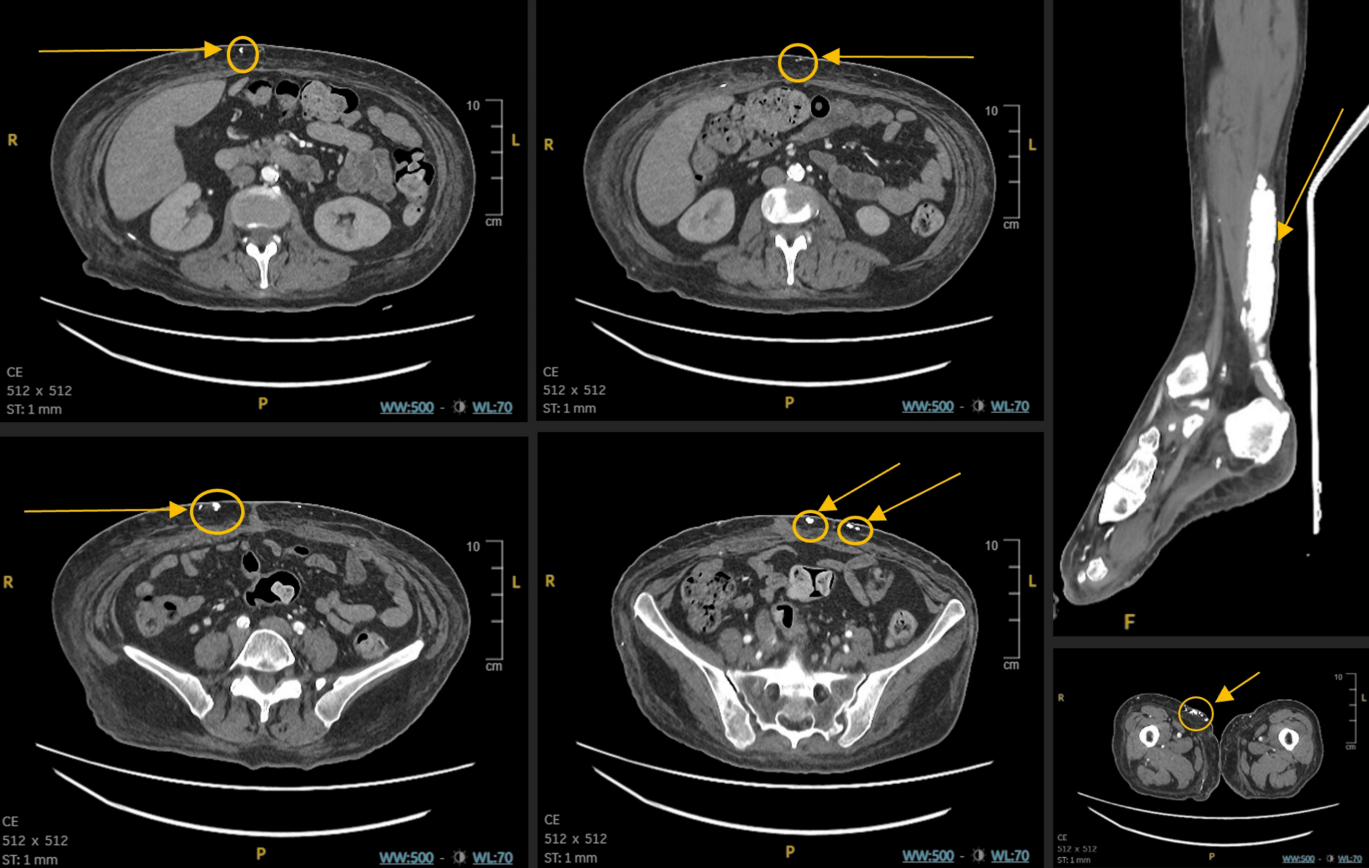
CT angiography of the lower limbs showing subcutaneous and intramuscular calcification. Please note the extensive calcification of the Achilles tendon.

Initial management included analgesia, aspirin, oral morphine, and intravenous antibiotics. Computed tomography (CT) angiography of the lower limbs showed oedema of subcutaneous fat, calcifications in the abdominal wall, gastrocnemius, and Achilles tendon, and similar calcifications in subcutaneous fat, muscle, and fascia bilaterally. These changes were suggestive of Dermatomyositis. There were also extensive atheromatous changes in the distal aorta, iliac, and leg arteries with significant bilateral superficial femoral artery narrowing and poor calf artery flow, as shown in Figure 3. In view of these findings, she was referred to the medical team for further work-up.

Her history included gradual, unintentional weight loss of approximately 80 kg over three years, without gastrointestinal, constitutional, respiratory, or genitourinary symptoms. She denied arthralgia, Raynaud’s phenomenon, dysphagia, skin thickening, or systemic features of malignancy such as haemoptysis, haematuria, vaginal bleeding, altered bowel habits, or early satiety. The nodules described had been present for three to four years, firm in consistency, and were not painful. On examination, multiple firm nodules were noted along the subcutaneous tissue, particularly around the elbows, causing skin tightness when straightening the limbs.

From the blood reports (as shown in Table 1), we can appreciate the following findings. Though renal insufficiency was present, we were not able to reliably distinguish whether it was acute or chronic, as there were no baseline blood tests to compare. Raised inflammatory parameters were in line with bilateral gangrenous first toes. Bone profile reveals hypercalcemia with suppressed parathyroid hormone, associated with low 25-hydroxy vitamin D levels, suspicious for malignancy; however, these parameters were normal on a repeat blood test. Thyroid tests in line with sick euthyroid syndrome were keeping in line with gangrenous infected toes. Hematinic revealed anaemia of chronic disease. Normal lipid profile and urate levels could rule out xanthomas or gouty tophi.

**Table 1:** Bloods revealed raised inflammatory markers, hypercalcaemia, low parathyroid and 25-hydroxy vitamin D levels. 25-OH-Vitamin D, 25-hydroxy vitamin D; ANA, anti-nuclear antibody; β2 microglobulin, beta-2 microglobulin; CRP, C-reactive protein; ENA, extractable nuclear antigen; ESR, erythrocyte sedimentation rate; Free T4, free thyroxine 4; GFR, glomerular filtration rate; IgA, immunoglobulin A; NXP2 antibody, nuclear matrix protein 2 antibody; PTH, parathyroid hormone; TIBC, total iron binding capacity; TSH, thyroid stimulating hormone

Test	Result	Normal range
Sodium	139	133-146 mmol/L
Potassium	3.6	3.5-5.3 mmol/L
Creatinine	135	45-84 μmol/L
GFR	41	≥ 90 mL/min/1.73 m²
CRP	68	0-5 mg/L
Haemoglobin	74	117-149g/L
White cell count	17.9	4.3-11.2 × 10^9^/L
Neutrophils	15.5	2.1-7.4 × 10^9^/L
Platelets	515	150-400 × 10^9^/L
Adjusted calcium	2.67	2.2-2.6 mmol/L
Phosphate	1.35	0.8-1.5 mmol/L
25-OH-vitamin D	11	>50 nmol/L
PTH	0.3	1.6-6.8 pmol/L
TSH	2.9	0.27-4.5 mU/L
Free T4	9.8	11-23 pmol/L
ANA	Multiple nuclear dot pattern	
ENA	Positive	
Anti-smooth muscle antibody	Weak positive	
β2 microglobulin	12.4	0.8-2.2 mg/L
IgA	6.4	0.7-4 g/L
NXP2 antibody	Positive	-
ESR	116	6-13 mm/hour
Serum iron	2.0	5.83-34.5umol/L
TIBC	37	45-70 μmol/L
Transferrin	1.47	2-3.6 μmol/L
Transferrin saturation	5	15-50%
Ferritin	155	20-130 µg/L
Serum urate	307	140-360 μmol/L
Lipid profile	Normal	-
Kappa	78.1	mg/L
Lamba	18	mg/L
Kappa/lamba ratio	Normal	-
Serum and urine protein electrophoresis	No monoclonal paraprotein or free light chains	
Urine protein creatinine ratio	81	<15 mg/mmol

After medical review, the following investigations were organized. Urine protein creatinine ratio, which was elevated, indicated a possible chronic kidney disease. A multiple myeloma screen ruled out plasma cell dyscrasia. A panel of tests for autoimmune myositis was organized in view of weight loss, intramuscular and subcutaneous calcifications, to look for one of the connective tissue diseases. Presence of the NXP2 antibody raised the suspicion of Dermatomyositis, and in adults, these antibodies are associated with malignancy and calcinosis cutis.

**Figure 4:**
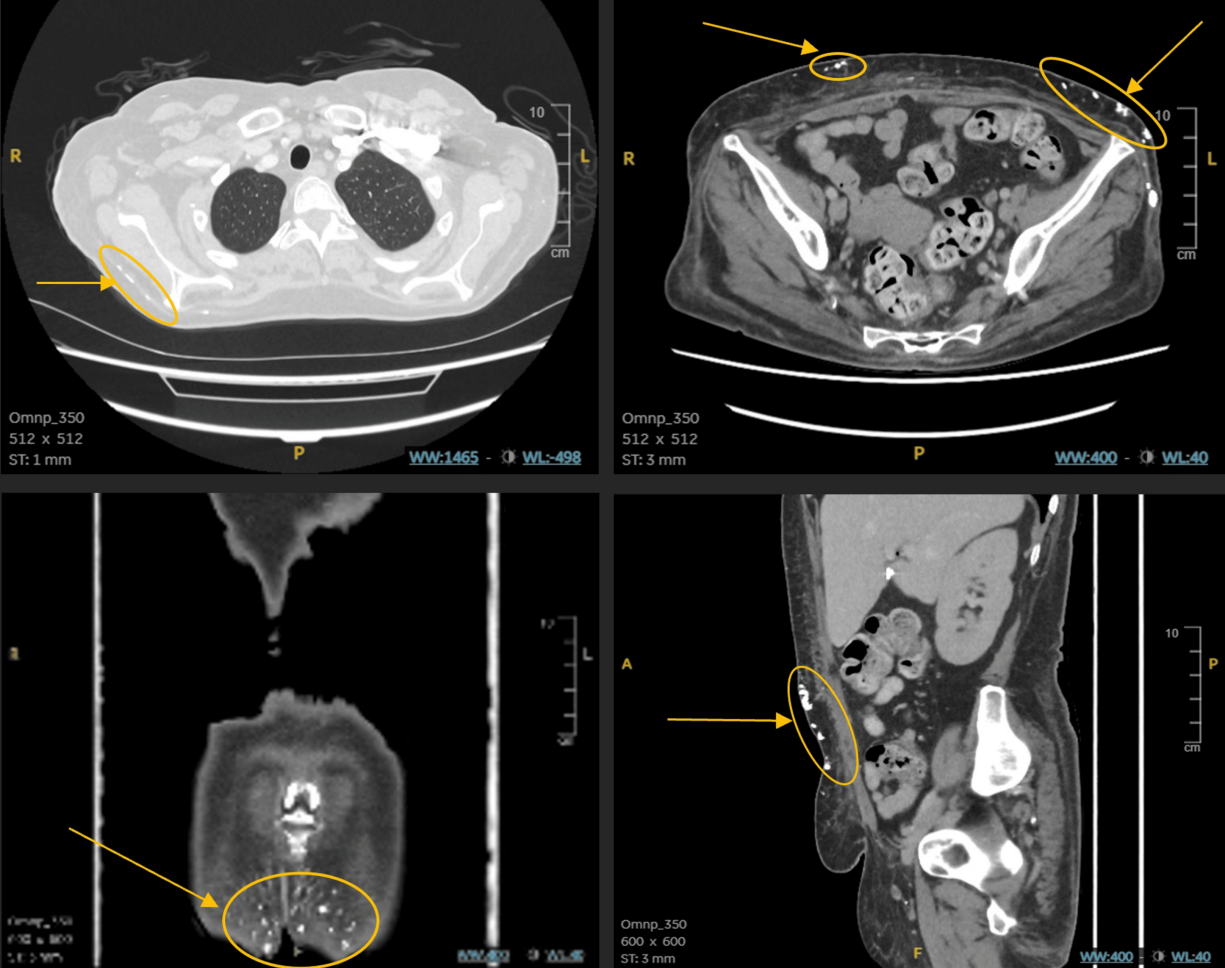
The neck-chest-abdomen-pelvis CT showing extensive calcinosis cutis affecting the chest, abdomen, and pelvic region.

A whole-body CT neck, chest, abdomen, and pelvis was done in view of the weight loss, hypercalcemia with suppressed PTH, and due to the possible association between dermatomyositis and malignancy. It reported multiple tiny areas of calcifications in subcutaneous fat with a few similar areas in muscles and intramuscular planes. Oedema of subcutaneous fat. No definitive aggressive lesion could be identified, as shown in Figure 4.

The renal team expressed their opinion that calciphylaxis is rare to happen before CKD stage 5 or dialysis. They advised seeking dermatology help for a biopsy from the gangrenous site. Dermatology agreed with the diagnosis of calcinosis cutis. However, did not pursue a biopsy. The rheumatology team identified no definite clinical features of dermatomyositis, such as Raynaud’s phenomenon, dysphagia, proximal muscle weakness, or classical cutaneous features. The distribution of nodules was consistent with calcinosis cutis. When these findings were integrated with imaging evidence and a strongly positive anti-NXP2 autoantibody, the overall picture aligned with a serologically and imaging-based diagnosis of amyopathic dermatomyositis. The vascular surgeon’s management: the right ABPI showed dorsalis pedis monophasic at 0.87, posterior tibial with no signal; left-sided ABPI was not completed due to pain. She underwent right superficial femoral and popliteal artery angioplasty with simultaneous toe debridement, followed by left-sided angioplasty at a subsequent admission. Postoperatively, both feet were warm with good capillary refill and intact motor and sensory function. The patient had no rest pain and no claudication pain. The clinical examination revealed progressive granulation of the amputation sites, with no signs of infection. Repeat Arterial Doppler clinical imaging confirmed improved perfusion of the right anterior tibial and popliteal arteries following angioplasty, while the left anterior tibial artery continued to show stable monophasic flow with chronic popliteal calcification.

In summary, this 43-year-old woman presented with bilateral gangrenous toes due to critical limb ischaemia, occurring in the context of extensive vascular calcification and long-standing firm subcutaneous nodules. Investigations revealed hypercalcaemia, suppressed PTH, chronic kidney disease, positive autoimmune serology including anti-NXP2 antibody, and imaging features consistent with calcinosis universalis. Malignancy screening was negative; however, given the strong association between anti-NXP2 dermatomyositis and malignancy, she remains under multidisciplinary follow-up.

The overall picture is most consistent with anti-NXP2-positive, clinically amyopathic dermatomyositis with calcinosis universalis and secondary critical limb ischaemia.

## Discussion

This patient, who presented with gangrenous toes, was treated as having critical limb ischemia. The presence of multiple skin nodules led us to investigate further. The presence of multiple subcutaneous nodules necessitated investigation beyond vascular pathology. Our differential diagnoses included eruptive xanthoma [7], primary dyslipidaemia [8], and chronic tophaceous gout [9-11], besides dystrophic calcinosis cutis and calciphylaxis. Although the presentation was similar to calciphylaxis, it was ruled out, as the patient’s renal function was stable and couldn’t account for it. Normal lipid and absence of yellowish papules helped exclude eruptive xanthoma [7] and primary dyslipidaemia [8]. Normal urate level and the absence of firm chalky nodules helped exclude gout-related nodulosis [9-11], which enabled calcinosis cutis as the leading possibility, particularly given the long-standing, firm, painless nodules and subsequent imaging evidence of widespread calcification.

With the significant weight loss, we were concerned about an occult malignancy. Once our literature search on calcinosis cutis showed that dermatomyositis was a common cause, we were able to connect the dots. Though the neck-chest-abdomen-pelvis CT could not identify any definitive aggressive lesion (malignancy), the presence of multiple tiny areas of calcifications in muscles and intramuscular planes gave us confidence to investigate for dermatomyositis. Further serologic evaluation revealed anti-NXP2 antibody positivity, which provided a pivotal diagnostic clue. Anti-NXP2 dermatomyositis is strongly associated with extensive calcinosis and carries an increased risk of malignancy, particularly in adult-onset disease [5]. Although the above-mentioned CT scan was negative for malignancy, it was decided to investigate the patient with PET-CT further, as Dermatomyositis with anti-NXP2 antibodies is highly associated with malignancy [1,12,13].

Calcinosis cutis is a recognized complication of dermatomyositis, though its frequency differs significantly between adults and children. In adult dermatomyositis, calcinosis occurs in approximately 30% of cases [1]. By contrast, in juvenile dermatomyositis (JDM), the reported incidence is much higher, ranging from 20% to 75% depending on disease duration and cohort studied [14]. Longer disease duration, delayed initiation of treatment, and specific autoantibody subsets such as anti-NXP2 have been associated with a greater likelihood of calcinosis development [14].

Classically, dermatomyositis presents with proximal muscle weakness and rashes. However, this patient lacked these features, consistent with the subset of clinically amyopathic or hypomyopathic dermatomyositis described in the literature [13]. Anti-NXP2 dermatomyositis without classic cutaneous or muscular signs has been reported, though infrequently, and is strongly associated with extensive calcinosis [13]. According to the 2017 EULAR/ACR classification criteria, patients without objective muscle weakness may still meet diagnostic thresholds when supported by characteristic imaging, serologic markers, and extramuscular manifestations [15]. In this case, the presence of anti-NXP2 antibodies and widespread calcinosis provided sufficient disease-specific features despite the absence of rash or proximal muscle involvement.

Dermatomyositis is classically regarded as an immune-mediated microangiopathy, in which complement-driven injury to small vessels leads to endothelial damage and tissue ischaemia; in anti-NXP2-positive disease, this mechanism has been linked to digital infarction and severe calcinosis [12,13]. In our patient, however, acute limb ischaemia occurred in the context of diffuse dystrophic calcinosis and marked arterial calcification on CT angiography, which demonstrated significant popliteal stenoses and peripheral arterial disease. This pattern suggests that the limb-threatening event was primarily driven by macrovascular atherosclerotic disease, likely exacerbated by chronic systemic inflammation and abnormal tissue repair related to dermatomyositis, rather than by small-vessel vasculopathy alone, and this presentation was unique. Although a contribution from immune-mediated microvascular occlusion cannot be excluded, the radiologic findings were more consistent with large-vessel pathology.

Importantly, this process is distinct from calciphylaxis, a severe vasculopathy defined by medial arteriolar calcification and thrombosis in the setting of advanced renal dysfunction and calcium-phosphate imbalance, with high associated mortality [3,4]. Our patient lacked the biochemical and clinical features typical of calciphylaxis, and her lesions were confined to the distal extremities, making a calciphylaxis-like mechanism unlikely. Taken together, the presentation is best explained by clinically amyopathic, anti-NXP2-positive dermatomyositis with extensive dystrophic calcinosis [1,14], occurring alongside and likely amplifying pre-existing large-vessel atherosclerotic disease to produce critical limb ischaemia. These atypical overlaps between autoimmune microangiopathy and macrovascular disease pose a diagnostic challenge, often delaying targeted therapy. Given the strong association between anti-NXP2 dermatomyositis and malignancy, these cases necessitate thorough baseline and interval oncologic surveillance, even when initial imaging is negative [12,13].

Imaging is a key adjunct in the evaluation of patients with dermatomyositis, as abnormalities are detected in a high proportion of patients, including those without overt muscle weakness. MRI of skeletal muscle is particularly sensitive, often showing oedema, fatty infiltration, and inflammation even in early or amyopathic cases, thereby guiding biopsy and monitoring disease activity. The characteristic MRI findings in patients with Dermatomyositis are usually subcutaneous high-signal intensity, fascial high-signal intensity, and a honeycomb pattern [16]. CT imaging is especially useful in detecting calcinosis cutis, vascular calcifications, and interstitial lung disease, all of which can accompany dermatomyositis. Reviews in JDM also highlight the utility of plain radiographs, ultrasound, and CT for documenting and tracking calcinosis, which may otherwise be underestimated clinically [13,14]. Furthermore, PET/CT has been employed in adults both to evaluate occult malignancy and to detect systemic inflammatory activity [13].

The management of calcinosis cutis in dermatomyositis remains challenging, as no single therapy is uniformly effective. The goals of treatment are to control the inflammation, relieve symptoms, prevent new calcium deposition, and manage complications such as ulceration or infection [1,14]. When calcinosis cutis occurs in the setting of malignancy, management should prioritize treatment of the underlying cancer, as disease control often parallels oncologic remission [12,13]. Immunosuppressive therapy may be continued cautiously alongside oncologic treatment to control myositis activity [13]. Adjunctive agents used for calcinosis (sodium thiosulfate, bisphosphonates, or diltiazem) can be considered for symptomatic relief, but emphasis remains on treating the neoplasm and controlling systemic inflammation [1,14].

In patients where malignancy is excluded, management focuses on long-term immunomodulation and symptom-directed therapy [1,14]. Maintain control of dermatomyositis with corticosteroids and steroid-sparing immunosuppressants [13].

Add calcium-modulating and anti-inflammatory drugs (diltiazem, bisphosphonates, sodium thiosulfate, or colchicine) as adjuncts when lesions are extensive or symptomatic [1,14].

When calcinosis causes significant pain, functional impairment, or recurrent ulceration, surgical excision may be considered for accessible lesions [1]. However, recurrence is common if underlying inflammation remains uncontrolled. Emerging local options include carbon dioxide laser ablation and extracorporeal shockwave therapy, which have been reported to reduce lesion size and pain in selected cases [1,14].

Given the complexity of presentations encompassing autoimmune microangiopathy and macrovascular disease, prompt evaluation supported by multidisciplinary input from vascular surgery, rheumatology, dermatology, radiology, and oncology is essential to guide appropriate management and reduce the risk of irreversible limb damage [17].

## Conclusions

This case highlights a rare and atypical presentation of anti-NXP2-positive dermatomyositis manifesting primarily with calcinosis cutis and critical limb ischemia, in the absence of classical cutaneous or muscular features. The detection of anti-NXP2 antibodies provided the crucial diagnostic clue. This case highlights the importance of oncological surveillance in patients with anti-NXP2 Dermatomyositis, even when initial imaging is negative. It underlines the importance of autoimmune myositis screening and comprehensive imaging in patients with calcinosis cutis, both of which were pivotal in arriving at the diagnosis in this atypical scenario. Current practice recommends baseline oncologic screening at diagnosis and annual surveillance for at least the first two to three years, as per recent consensus recommendations. Dermatomyositis, although typically characterized by microvascular involvement, can, in rare instances, present with large-vessel calcification leading to limb-threatening complications. Early recognition and appropriate diagnostic workup, coordinated with multidisciplinary care involving vascular surgery, rheumatology, dermatology, oncology, and radiology, are essential for atypical presentations to prevent irreversible outcomes such as amputations.
